# Diagnostic Potential of Cross-Questionnaire Analysis for Depression and Memory Disorders Using Machine Learning Techniques

**DOI:** 10.1192/j.eurpsy.2025.476

**Published:** 2025-08-26

**Authors:** M. Balkoudi, N. Arnaoutoglou, K. Fountoulakis, A. Saitis, G. Deretzi, I. Diakogiannis, D. Hristu-Varsakelis

**Affiliations:** 1 Department of Applied Informatics, University of Macedonia; 2 3rd Dpt Of Psychiatry; 3 1st Dpt Of Psychiatry, Aristotle University Thessaloniki; 4 Neurology Clinic, Papageorgiou General Hospital, Thessaloniki, Greece

## Abstract

**Introduction:**

Research shows a strong correlation between depression and memory disorders, suggesting the potential for cross-questionnaire data use in automated diagnostic systems. This study explores whether the Prospective and Retrospective Memory Questionnaire (PRMQ) can identify depressive symptoms and if the ZUNG Self-Rating Depression Scale (SDS) can predict memory-related disorders.

**Objectives:**

To evaluate the effectiveness of using questionnaires intended for one mental disorder to diagnose another through machine learning models on data from a large-scale self-assessment online questionaire.

**Methods:**

The study is part of the Memory and Depression Study: MANDY, conducted by the 1st Department of Psychiatry and the Department of Neurology of Papageorgiou Hospital, Thessaloniki, Greece. Data from 3340 participants were collected via an online survey containing the PRMQ, SDS, demographic data, and health-related questions. Four predictive tasks were designed: two for predicting depression using memory responses (D-from-M score and class) and two for predicting memory disorders using depression responses (M-from-D score and class). Machine learning models including LightGBM, AdaBoost, Support Vector Machines, and Logistic Regression were evaluated. Performance metrics included precision, recall, F1-score, and AUC-ROC (Figure 1).

**Results:**

The LightGBM classifier was the top-performing model for the D-from-M class prediction task, achieving a precision of 0.75563, recall of 0.79125, an F1-score of 0.77303, and an AUC-ROC of 0.79319 on the test set (Table 1). This indicates a strong predictive capability for diagnosing depression from memory-related responses. The AdaBoost classifier had similar performance but was slightly inferior to LightGBM. For the M-from-D class task, the class imbalance (memory disorder prevalence at 5%) was a significant challenge. The best model, a Support Vector Classifier with ADASYN resampling, achieved a precision of 0.6, recall of 0.375, an F1-score of 0.46154, and an AUC-ROC of 0.86218. However, its performance was notably lower than LightGBM in predicting depression.

**Image 1:**

**Figure:**
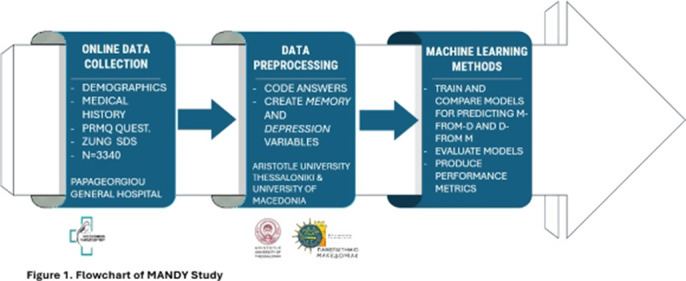

**Image 2:**

**Figure:**
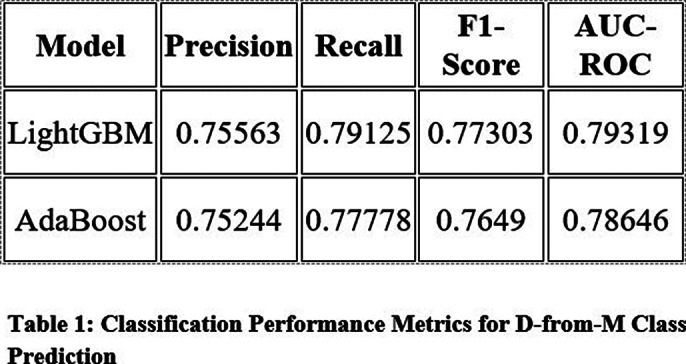

**Conclusions:**

The PRMQ, combined with specific demographic and health-related questions, showed promise in predicting depression, with the LightGBM classifier as the best overall model. This underscores the potential for cross-questionnaire data utilization for diagnosing depression. Conversely, predicting memory disorders using the SDS was less effective, indicating the need for more targeted diagnostic tools. Future research should include neurocognitive and biomarker data to enhance diagnostic accuracy for memory-related conditions.

**Disclosure of Interest:**

None Declared

